# Prognostic impact of positive peritoneal washing cytology in surgically treated cervical cancer

**DOI:** 10.1097/MD.0000000000051356

**Published:** 2026-11-06

**Authors:** Qianwen Zhang, Xiaoqin Li, Qi Wang, Zhiqian Lin, Tao Wang

**Affiliations:** a Department of Gynecologic Oncology, Sun Yat-sen University Cancer Center Gansu Hospital, Lanzhou, Gansu Province, China; b Department of Pathology, Sun Yat-Sen University Cancer Center Gansu Hospital, Lanzhou, Gansu Province, China; c Translational Medicine Research Center, Sun Yat-Sen University Cancer Center Gansu Hospital, Lanzhou, Gansu, China.

**Keywords:** Cervical Cancer, Lymph node metastasis, Matrix metalloproteinase-9, Peritoneal Cytology, Survival analysis, Vascular endothelial growth factor

## Abstract

Positive peritoneal washing cytology is an uncommon but potentially clinically relevant finding in cervical cancer. This retrospective cohort study evaluated its association with recurrence, survival, and metastasis-related biomarkers in patients undergoing open surgery for cervical cancer between 2018 and 2023. A total of 120 patients were classified as peritoneal washing cytology-positive (n = 32) or cytology-negative (n = 88). Baseline clinicopathological characteristics were broadly comparable between groups. Kaplan–Meier analysis demonstrated poorer survival among patients with positive cytology. The estimated 5-year overall survival was 43.7% (95% CI, 26.8–59.4%) in the cytology-positive group and 67.9% (95% CI, 55.2–77.8%) in the cytology-negative group, whereas the corresponding 5-year progression-free survival estimates were 36.5% (95% CI, 20.5–52.6%) and 62.4% (95% CI, 49.8–72.8%), respectively. Recurrence was more frequent among cytology-positive patients, particularly distant-organ recurrence. Serum VEGF and tissue MMP-9 levels were higher in the cytology-positive group. In multivariable analysis, positive peritoneal washing cytology remained associated with poorer overall survival after adjustment for selected clinicopathological variables. A combined VEGF/MMP-9 model showed exploratory discrimination for lymph-node metastasis, although the model requires internal and external validation. These findings suggest that positive peritoneal washing cytology may identify a subgroup with adverse outcomes after surgery, but prospective and externally validated studies are required before clinical application.

## 1. Introduction

Cervical cancer remains a major global health burden, particularly in low- and middle-income countries. According to GLOBOCAN 2020 estimates, cervical cancer continues to rank among the most commonly diagnosed malignancies and leading causes of cancer-related mortality in women worldwide.^[1,2]^ Although human papillomavirus vaccination and cervical cancer screening have contributed to reductions in disease incidence, the prognosis of patients with recurrent or advanced disease remains unfavorable.^[3,4]^ The 2018 International Federation of Gynecology and Obstetrics (FIGO) staging system incorporates major anatomical and pathological factors relevant to prognosis; nevertheless, considerable heterogeneity in recurrence and survival remains among patients with similar clinicopathological characteristics.^[5]^ These observations highlight the need to identify additional factors that may improve risk stratification in cervical cancer.

Peritoneal washing cytology is not part of the FIGO staging definition for cervical cancer, and published evidence regarding its prognostic significance is limited and heterogeneous.^[6]^ A cervical cancer-specific study by Ditto et al evaluated peritoneal cytology after radical hysterectomy, and a meta-analysis by Yoon et al found associations between positive peritoneal cytology, recurrence, and lymph-node metastasis. More recently, Takigawa et al reported that positive peritoneal washing cytology was relatively frequent in gastric-type cervical adenocarcinoma and was associated with adverse pathological features and poorer outcomes in selected patients.^[7,8]^ These data support investigation of peritoneal washing cytology as a risk marker while also emphasizing histologic and selection effects.

The biological interpretation of peritoneal tumor cells should be cautious. Vascular endothelial growth factor expression has been associated with lymph-node metastasis in cervical cancer, and matrix metalloproteinase-9 expression has been associated with invasive pathological features and prognosis. However, cross-sectional differences in biomarker levels cannot establish that peritoneal tumor-cell shedding causes angiogenesis or matrix remodeling. In addition, literature on minimally invasive radical hysterectomy suggests that intraperitoneal tumor exposure may influence recurrence patterns, but the present cohort underwent open surgery and does not directly test pneumoperitoneum-related mechanisms.^[9–11]^

Lymph-node metastasis is an important determinant of recurrence and survival in cervical cancer. Imaging-based evaluation, including magnetic resonance imaging, is commonly used for preoperative assessment of nodal disease, although diagnostic accuracy remains imperfect.^[12,13]^ Recent studies have therefore explored complementary pathological and molecular factors associated with lymph-node metastasis.^[14,15]^ MMP-9 expression, in particular, has been associated with pelvic and para-aortic lymph-node metastasis in cervical cancer.^[15]^ Nevertheless, the relationships among peritoneal washing cytology status, VEGF and MMP-9 levels, and lymph-node involvement have not been sufficiently characterized.

Accordingly, this study had 3 objectives: to evaluate the association between peritoneal washing cytology and overall/progression-free survival; to describe recurrence patterns using clinically appropriate full-cohort denominators; and to explore associations of cytology status with serum vascular endothelial growth factor and tissue matrix metalloproteinase-9 and their discriminatory performance for lymph-node metastasis.

## 2. Materials and methods

### 2.1. Study population

This study was approved by the Ethics Committee of Sun Yat-sen University Cancer Center Gansu Hospital. This was a retrospective cohort study including 120 patients with cervical cancer who underwent open surgery at Sun Yat-sen University Cancer Center Gansu Hospital between May 2018 and June 2023, meeting the inclusion and exclusion criteria. Data were derived from the hospital’s electronic medical record system (EMR) and pathological database. The study was approved by the hospital ethics committee (approval number: A2024 04080017), and informed consent was waived by the Ethics Committee.

### 2.2. Inclusion criteria

1. pathologically confirmed cervical cancer after surgery;
2. intraoperative peritoneal washing/peritoneal fluid collection performed according to the institutional procedure, with adequate specimen volume and available cytological evaluation results;
3. complete key clinicopathological data, including at minimum FIGO stage category, tumor diameter, and lymph-node metastasis status; and
4. adequate outcome information for survival analysis. Adequate follow-up was defined as either documented study events during follow-up or, for patients who remained alive and event-free, at least 36 months of available follow-up.

### 2.3. Exclusion criteria

1. concurrent other primary malignant tumors;
2. inadequate or unsuccessful peritoneal washing cytology assessment;
3. missing key clinicopathological data; or
4. insufficient follow-up information for outcome analysis, including early loss to follow-up without documented outcome events. In the original dataset, patients with follow-up shorter than 6 months and without analyzable outcome information were excluded on this basis (Fig. 1).

**Figure 1.**
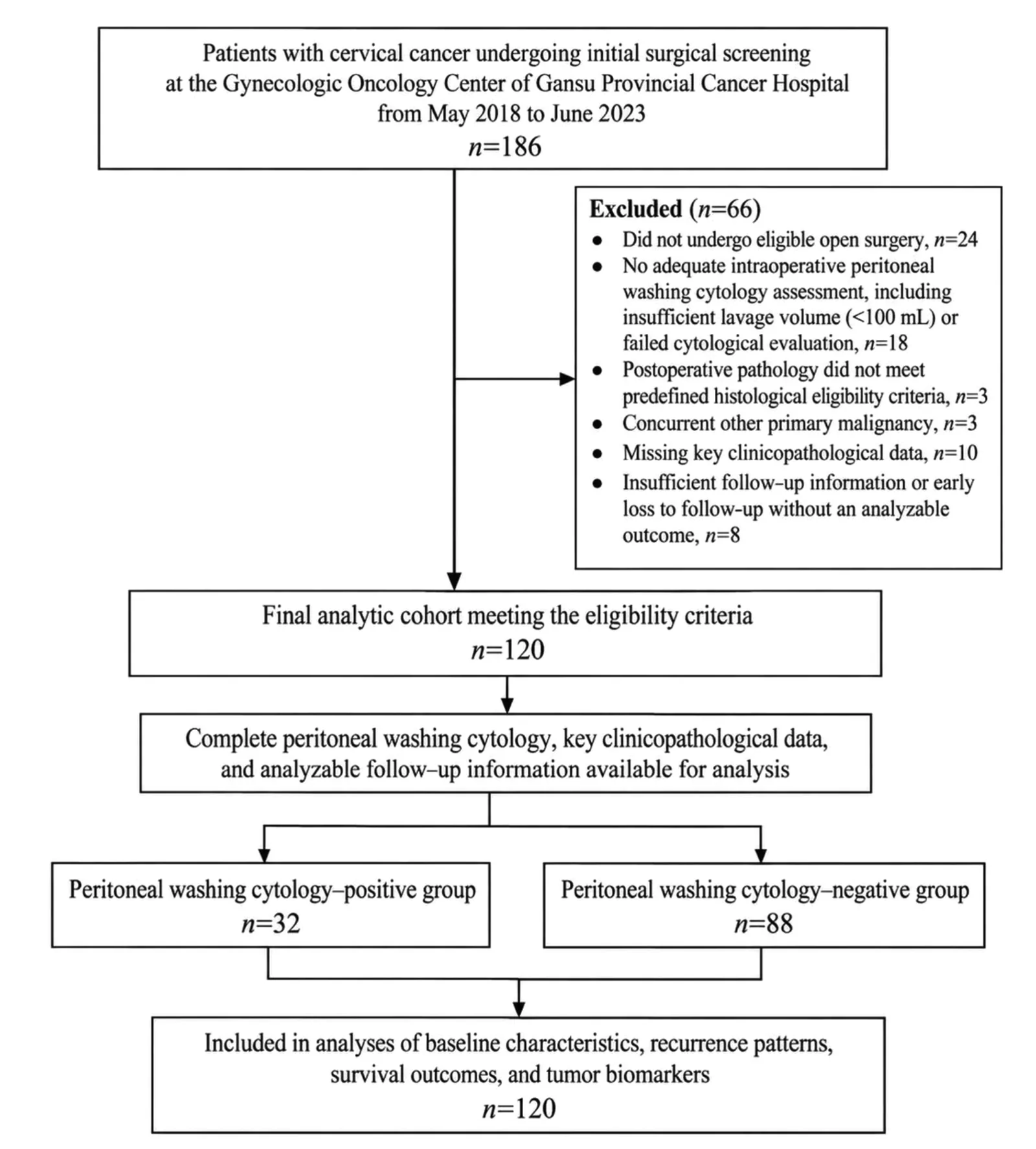
Flowchart of the study population selection.

### 2.4. Grouping criteria

According to intraoperative peritoneal cytological evaluation results, patients were divided into the positive peritoneal cytolog group (Cyt^+^ group), where malignant tumor cells were detected by ThinPrep liquid-based cytology (n = 32), and the negative peritoneal cytolog group (Cyt^-^ group), where no malignant cells were detected by ThinPrep liquid-based cytology (n = 88). Cytological results in both groups were reviewed by 2 chief physicians, and inconsistent cases were confirmed by pathological consultation. There were no statistically significant differences in key characteristics such as age, FIGO stage, and tumor diameter between the Cyt^+^ and Cyt^-^ groups (*P* > .05).

### 2.5. Sample size calculation

Sample size estimation was performed using PASS 15.0 software, with overall survival (OS) as the primary endpoint. Referencing previous studies^[16]^ and pilot experiment results, the expected 5-year OS rates were 40% for the Cyt^+^ group and 60% for the Cyt^-^ group, with a two-sided α = 0.05 and power 1-β = 0.80. Based on the Log-rank test sample size formula and considering a 20% dropout rate, a total of 120 cases were included (32 in the Cyt^+^ group and 88 in the Cyt^-^ group). This sample size meets the statistical power for detecting survival differences between groups (effect size d = 0.55) and multivariate Cox regression analysis (VIF < 10).

### 2.6. Research methods

#### 2.6.1. Peritoneal cytological evaluation

Sample Collection: After entry into the abdominal cavity and before tumor resection, 200 mL of sterile normal saline was distributed through the pelvis, including the pouch of Douglas and bilateral paracolic gutters. At least 100 mL was aspirated. The specimen was processed for liquid-based cytology. Slides were fixed, Papanicolaou stained, and independently reviewed by 2 senior pathologists; discrepant cases were resolved by departmental consultation. Patients were classified as cytology positive when malignant cells were identified and cytology negative when malignant cells were not identified.

Liquid-Based Cytology: The remaining peritoneal washing fluid sample was centrifuged at 2000 r/min for 15 minutes. The pellet was added to ThinPrep preservation fluid (lot number: 289182), and thin-layer smears were prepared using a Hologic 3000 automated slide processor. Smears were fixed with 95% ethanol for 15 minutes and stained with Papanicolaou (hematoxylin for 5 minutes, eosin for 3 minutes). Stained smears were independently reviewed by 2 senior pathologists, and discrepant cases were confirmed by departmental consultation. Additionally, remaining peritoneal washing fluid was centrifuged at 4°C, 3000 rpm for 15 minutes, and cell-free supernatants were aliquoted into sterile cryotubes at 0.5 mL/tube, stored at −80°C to avoid repeated freeze-thaw cycles, and used for subsequent VEGF testing.

### 2.7. MMP-9 and VEGF detection

Sample Preparation: Preoperative fasting venous blood (3 mL) was centrifuged at 3000 rpm for 10 minutes, and serum was stored at −80°C for VEGF testing. Postoperative fresh tumor tissue (0.5 g) was added to pre-cooled lysis buffer with protease inhibitors, homogenized in an ice-water bath, and centrifuged at 12,000 rpm for 20 minutes to collect supernatants for MMP-9 testing.

ELISA Assay: Quantitative detection was performed using enzyme-linked immunosorbent assay (ELISA) kits (R&D Systems, USA; VEGF kit lot DVE00, MMP-9 kit lot M9000). Procedures included: Pre-coated microplate wells with antihuman VEGF/MMP-9 monoclonal antibodies, equilibrated at room temperature for 30 minutes; Addition of 50 μL standards (0, 25, 50, 100, 200, 400, 800, 1000 pg/mL) or samples, incubated at 37°C for 2 hours, washed 3 times, followed by 50 μL HRP-conjugated avidin working solution, incubated at 37°C for 1 hour; and addition of 100 μL TMB substrate, reacted at 37°C in the dark for 15 minutes, terminated with 50 μL stop solution (2M H_2_SO_4_), and absorbance measured at 450 nm using a microplate reader (Thermo Scientific Multiskan FC). Concentrations were calculated from standard curves (VEGF: ng/mL; MMP-9: pg/mg protein).

### 2.8. Observational parameters

Clinical Data Collection: Baseline Characteristics: Age (precise to years, continuous variable), body mass index (BMI, kg/m^2^), and underlying diseases. Pathological Characteristics: Pathological grade (well-moderately differentiated vs poorly differentiated); tumor type (squamous cell carcinoma vs adenocarcinoma/ adenosquamous carcinoma); tumor diameter (postoperative pathological measurement, < 4 cm vs ≥ 4 cm); stromal invasion depth (superficial < 1/2 stromal invasion depth vs deep ≥ 1/2 stromal invasion depth); lymphovascular space invasion (LVSI, yes/no); pelvic lymph node metastasis (pathologically confirmed ≥ 1 metastatic node after lymphadenectomy, yes/no). Treatment-Related Data: Surgical approach (simple hysterectomy vs radical hysterectomy + pelvic lymph node dissection); FIGO stage (2018 revised version, stage I vs II); preoperative SCC level (normal < 1.5 ng/mL vs elevated ≥ 1.5 ng/mL). Survival Data: Overall survival (OS, days from surgery to death or last follow-up); progression-free survival (PFS, days from surgery to recurrence, metastasis, death, or last follow-up, whichever occurred first); recurrence characteristics including recurrence time and site (para-aortic/pelvic recurrence, distant organ recurrence [lung/liver/bone, etc.], or combined pelvic + distant recurrence).

### 2.9. Follow-up protocol

Follow-up was conducted via outpatient reviews (70%) and telephone follow-up (30%). The frequency was every 3 months for the first 2 years (including pelvic MRI/CT and tumor marker SCC testing), every 6 months for years 3 to 5 (chest-abdomen-pelvic CT), and annually after 5 years (primarily telephone confirmation of survival status).

Overall survival was defined as time from surgery to death from any cause or last follow-up. Progression-free survival was defined as time from surgery to first recurrence, metastasis, death, or last follow-up. Recurrence was categorized as para-aortic/pelvic, distant organ, or combined pelvic + distant. For site-specific recurrence analyses, the denominator was the entire cytology group rather than only the subgroup of patients who recurred.

### 2.10. Observational indexes

Baseline Characteristics: Age (years), pathological grade (well + moderately differentiated vs poorly differentiated), tumor type (squamous cell carcinoma vs adenocarcinoma/ adenosquamous carcinoma), tumor diameter (< 4cm vs ≥ 4 cm), stromal invasion depth (superficial < 1/2 stromal invasion depth vs deep ≥ 1/2 stromal invasion depth), LVSI (yes/no), FIGO stage (2018 version, I vs II), surgical approach (simple hysterectomy vs radical resection), lymph node dissection (yes/no), and preoperative SCC level (normal < 1.5ng/mL vs elevated ≥ 1.5ng/mL). Survival Outcomes: OS (time from surgery to all-cause death, censored at last follow-up for survivors); PFS (time from surgery to recurrence, metastasis, or death, censored at last follow-up for nonevents), with event times recorded precisely for survival analysis. Recurrence Characteristics: Recurrence rate (%) = (number of recurrent patients/total cases) × 100%; recurrence sites (para-aortic/pelvic, distant organs [lung/liver/bone, etc.], or combined pelvic + distant). Diagnostic Efficacy Evaluation: Using pathology-confirmed LN metastasis (≥ 1 node) as gold standard: ROC analysis for serum VEGF and tissue MMP-9 (AUC, optimal cutoff, sensitivity/specificity).

### 2.11. Statistical analysis

Continuous variables were summarized as mean ± standard deviation or median (interquartile range), as appropriate. Categorical variables were summarized as n (%). Baseline balance was described using standardized mean differences in addition to hypothesis tests. Overall survival and progression-free survival were analyzed separately by Kaplan–Meier methods and compared with separate log-rank tests. Median follow-up was estimated by the reverse Kaplan–Meier method. Kaplan–Meier estimates at 36, 48, and 60 months are reported with 95% confidence intervals, and median survival is reported only when the survival function reached 0.50. Numbers at risk are displayed below each survival curve. Site-specific recurrence comparisons used the full cytology-group denominators; Fisher’s exact test was used when cell counts were small. For biomarker analyses, unadjusted group comparisons used the Mann–Whitney *U* test. Exploratory adjusted log-linear models included cytology status, broad FIGO stage, lymph-node metastasis, tumor diameter, grade, lymphovascular space invasion, cervical stromal invasion depth, histology, and preoperative squamous cell carcinoma antigen category. For overall survival, the primary adjusted Cox model included cytology status, broad FIGO stage, and lymph-node metastasis; additional univariable estimates are shown for other available prognostic variables. For lymph-node metastasis, an exploratory logistic model used serum vascular endothelial growth factor and tissue matrix metalloproteinase-9 as continuous predictors. The fitted equation was logit(P) = −5.3419 + 0.02510 × serum vascular endothelial growth factor + 0.02102 × tissue matrix metalloproteinase-9. A sensitivity model additionally included cytology status. Discrimination was evaluated by area under the receiver operating characteristic curve. Confidence intervals and paired model comparisons were obtained by bootstrap resampling, and sensitivity/specificity confidence intervals were calculated using binomial methods. Numerical differences in area under the curve were not interpreted as statistically superior without a supported pairwise comparison. All tests were two-sided.

## 3. Results

### 3.1. Comparison of baseline patient characteristics

The analytic cohort contained 120 patients: 32 (26.7%) with positive peritoneal washing cytology and 88 (73.3%) with negative cytology. The full histological distribution was 93/120 squamous cell carcinomas, 20/120 adenocarcinomas, and 7/120 adenosquamous carcinomas. Broad FIGO stage, tumor size, histology, and surgical procedure were similar between groups, but lymph-node metastasis was more frequent in the cytology-positive group (16/32, 50.0%) than in the cytology-negative group (26/88, 29.5%), standardized mean difference 0.43. Deep cervical stromal invasion also showed an imbalance (standardized mean difference 0.37).

Among the 120 patients analyzed, the cytology positivity was 14/56 (25.0%) in broad stage I and 18/64 (28.1%) in broad stage II; by histology subtype, the rate was 24/93 (25.8%) for squamous cell carcinoma, 6/20 (30.0%) for adenocarcinoma, and 2/7 (28.6%) for adenosquamous carcinoma (Table 1).

**Table 1:** Comparison of baseline characteristics.

Variables	Cyt^+^ group (n = 32)	Cyt^−^ group (n = 88)	Statistical value	*P* value	Absolute standardized difference
Age (years, $\bar{x}\pm s$)	52.34 ± 8.21	50.89 ± 9.47	t = 0.782	.436	0.16
BMI (kg/m^2^, $\bar{x}\pm s$)	24.16 ± 3.52	23.75 ± 3.89	t = 0.531	.597	0.11
FIGO stage [n (%)]			χ^2^ = 0.216	.642	0.08
Stage I	14 (43.75)	42 (47.73)			
Stage II	18 (56.25)	46 (52.27)			
Tumor diameter [n (%)]			χ^2^ = 0.103	.748	0.07
<4 cm	12 (37.50)	36 (40.91)			
≥4 cm	20 (62.50)	52 (59.09)			
Pathological grade [n (%)]			χ^2^ = 1.842	.175	0.25
Well/Moderately differentiated	10 (31.25)	38 (43.18)			
Poorly differentiated	22 (68.75)	50 (56.82)			
LVSI Positive [n (%)]	25 (78.13)	58 (65.91)	χ^2^ = 1.782	.182	0.27
Preoperative SCC ≥ 1.5 ng/mL [n (%)]	26 (81.25)	65 (73.86)	χ^2^ = 0.704	.401	0.18
Histology			χ^2^ = 0.162	.922	
Squamous cell carcinoma	24 (75.0%)	69 (78.4%)			0.08
Adenocarcinoma	6 (18.8%)	14 (15.9%)			0.06
Adenosquamous carcinoma	2 (6.2%)	5 (5.7%)			0.02
Lymph-node metastasis	16 (50.0%)	26 (29.5%)	0.43	.038	0.43
Deep cervical stromal invasion ≥ 1/2	21 (65.6%)	42 (47.7%)	0.37	.083	0.37

Continuous variables are presented as mean ± standard deviation, compared using independent samples *t*-test; categorical variables are presented as frequency (percentage), compared using chi-squared test.

BMI = body mass index, LVSI = lymphovascular space invasion.

### 3.2. Survival analysis outcomes

There were 19 deaths and 13 overall-survival censored observations in the cytology-positive group, compared with 34 deaths and 54 censored observations in the cytology-negative group. For progression-free survival, there were 21 events and 11 censored observations in the cytology-positive group and 38 events and 50 censored observations in the cytology-negative group. Reverse Kaplan–Meier median follow-up was 70.1 months overall (65.6 months in the cytology-positive group and 71.6 months in the cytology-negative group).

The overall-survival curves differed significantly (log-rank χ^2^ = 9.080, *P* = .003), as did the progression-free-survival curves (log-rank χ^2^ = 6.036, *P* = .014). Median overall survival was 52.1 months in the cytology-positive group and was not reached in the cytology-negative group. Median progression-free survival was 38.1 months in the cytology-positive group and was not reached in the cytology-negative group (Table 2) (Figs. 2 and 3).

**Table 2:** Comparison of survival outcomes.

Group	Outcome	Events/ censored	36-month estimate (95% CI)	48-month estimate (95% CI)	60-month estimate (95% CI)	Median, months
Positive peritoneal washing cytology	Overall survival	19/ 13	75.0% (56.2–86.6)	59.4% (40.5–74.0)	40.6% (23.8–56.8)	52.1
Positive peritoneal washing cytology	Progression-free survival	21/ 11	65.6% (46.6–79.3)	43.8% (26.5–59.8)	34.4% (18.8–50.6)	38.1
Negative peritoneal washing cytology	Overall survival	34/ 54	100.0% (100.0–100.0)	88.6% (79.9–93.7)	61.4% (50.4–70.6)	Not reached
Negative peritoneal washing cytology	Progression-free survival	38/ 50	78.4% (68.3–85.6)	62.5% (51.5–71.7)	56.8% (45.8–66.4)	Not reached

Survival curves compared using Log-rank test.

OS = overall survival, PFS = progression-free survival.

**Figure 2.**
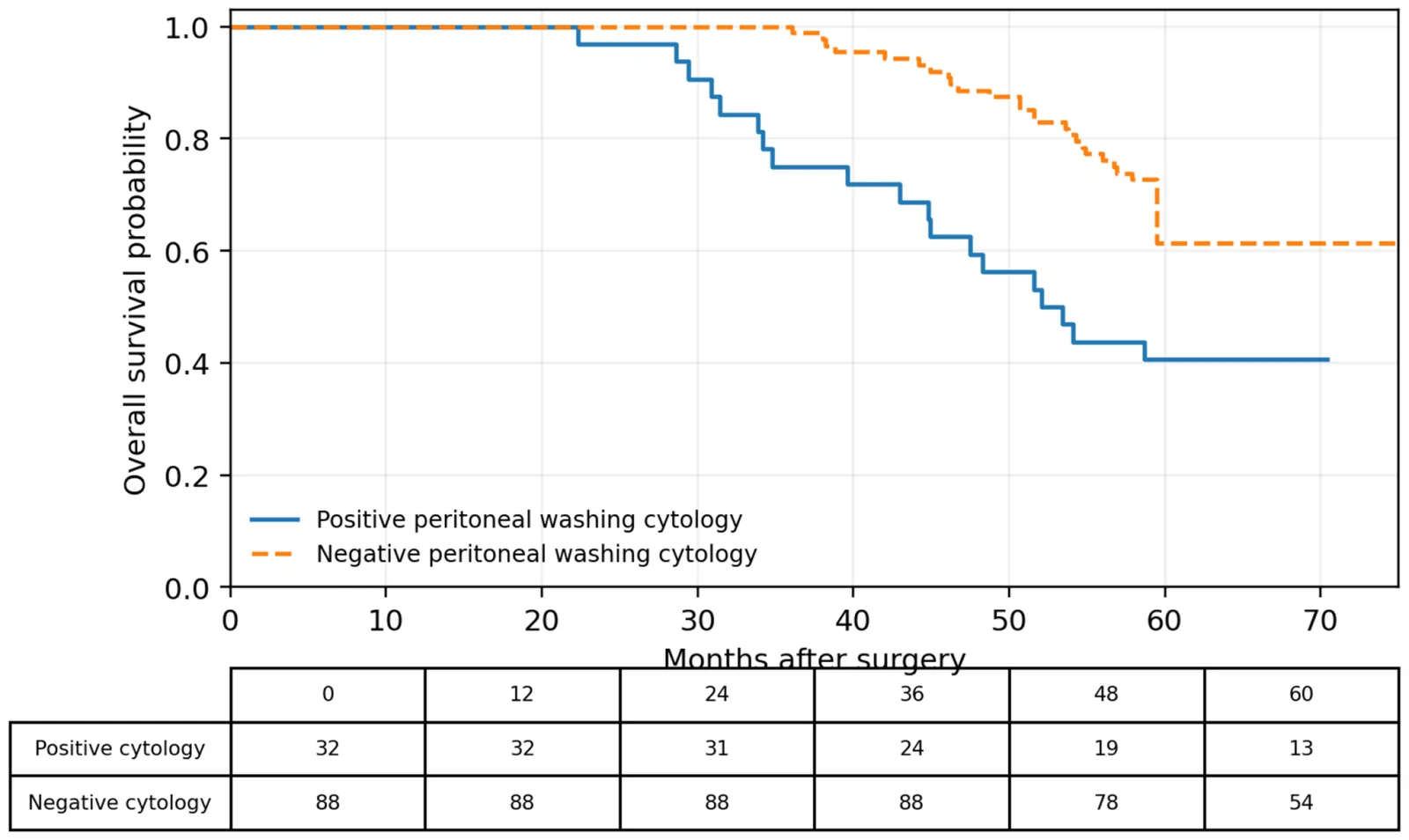
Kaplan–Meier survival curves according to peritoneal washing cytology status. (A) Overall survival. (B) Progression-free survival. Numbers at risk are shown below each survival curve. Differences between groups were assessed using the log-rank test.

**Figure 3.**
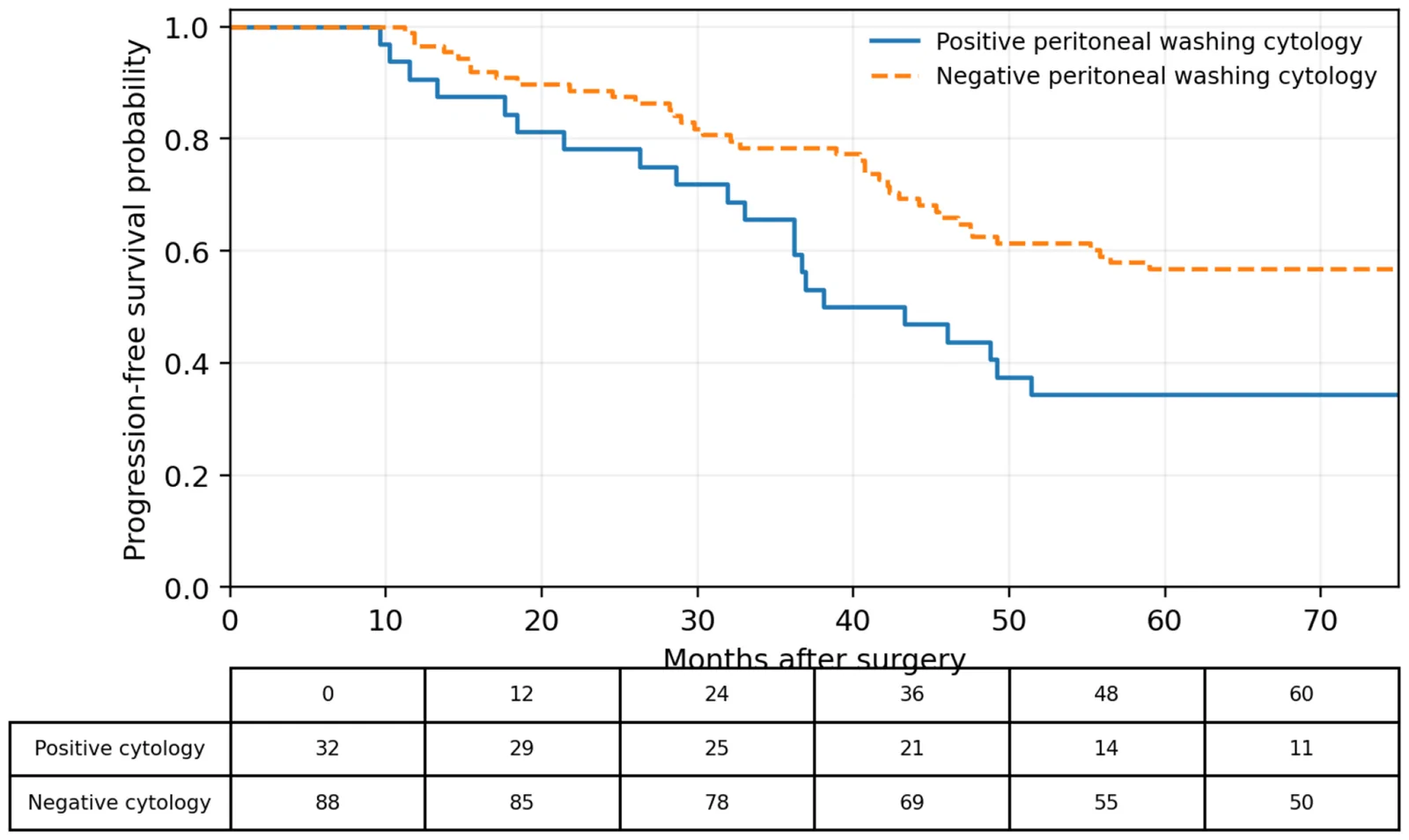
Kaplan–Meier curve for progression-free survival, with numbers at risk beneath the curve.

### 3.3. Recurrence patterns

Recurrence occurred in 19/32 (59.4%) cytology-positive patients and 28/88 (31.8%) cytology-negative patients. Using the clinically relevant full-group denominators, distant-organ recurrence occurred in 7/32 (21.9%) vs 3/88 (3.4%), whereas para-aortic/pelvic recurrence and combined pelvic + distant recurrence did not show statistically significant differences (Table 3).

**Table 3:** Recurrence risk by site using full cytology-group denominators [n (%)].

Recurrence site	Cyt^+^ group (n = 32)	Cyt^−^ group (n = 88)	*P* value
Any recurrence	19/32 (59.4%)	28/88 (31.8%)	.010
Para-aortic/pelvic recurrence	6/32 (18.8%)	12/88 (13.6%)	.565
Distant-organ recurrence	7/32 (21.9%)	3/88 (3.4%)	.004
Combined pelvic + distant recurrence	6/32 (18.8%)	13/88 (14.8%)	.582

Recurrence rates were compared using the chi-squared test; Bonferroni correction was applied for multiple comparisons of recurrence sites (α = 0.0167), with *P* < .0167 considered statistically significant.

### 3.4. Serum VEGF and tissue MMP-9 levels

The Cyt^+^ group demonstrated significantly elevated serum VEGF (median: 98.25 ng/mL, IQR: 84.36–116.74 ng/mL vs 72.83 ng/mL, IQR: 58.42–89.17 ng/mL; Z = 4.832, *P* < .001) and tissue MMP-9 levels (median: 142.60 pg/mg protein, IQR: 118.75–169.35 pg/mg vs 102.40 pg/mg protein, IQR: 80.55–124.85 pg/mg; Z = 5.127, *P* < .001). Non-normal distributions were confirmed by Shapiro–Wilk testing (W = 0.924, *P* < .05), suggesting that peritoneal cytology positivity is associated with enhanced angiogenic and matrix-remodeling activity (Table 4). Serum vascular endothelial growth factor and tissue matrix metalloproteinase-9 were higher in the cytology-positive group in unadjusted analyses. In exploratory log-linear models adjusted for broad FIGO stage, lymph-node status, tumor diameter, grade, lymphovascular space invasion, cervical stromal invasion depth, histology, and preoperative squamous cell carcinoma antigen category, cytology positivity remained associated with higher biomarker levels. These models support an association but do not establish a causal biological pathway.

**Table 4:** Comparison of VEGF and MMP-9 levels between groups.

Indicators	Cyt^+^ group (n = 32)	Cyt^−^ group (n = 88)	Unadjusted *P* value	Adjusted cytology association
VEGF (ng/mL)	98.25 (84.36–116.74)	72.83 (58.42–89.17)	.001	Adjusted geometric mean ratio 1.18 (95% CI 1.02–1.37), *P* = .026
Tissue MMP-9 (pg/mg protein)	142.60 (118.75–169.35)	102.40 (80.55–124.85)	.002	Adjusted geometric mean ratio 1.16 (95% CI 1.00–1.34), *P* = .045

Data are presented as median (interquartile range); non-normal distribution confirmed by Shapiro–Wilk test (W = 0.924, *P* < .05); intergroup comparisons performed using Mann–Whitney *U* test.

MMP-9 = Matrix Metalloproteinase-9, VEGF = Vascular Endothelial Growth Factor.

### 3.5. Cox regression analysis for overall survival

In univariable Cox analysis, cytology positivity and lymph-node metastasis were associated with mortality. In the prespecified three-variable model containing cytology status, broad FIGO stage, and lymph-node metastasis, cytology positivity remained associated with mortality (adjusted hazard ratio 2.20, 95% confidence interval 1.25–3.87, *P* = .007); lymph-node metastasis was borderline statistically significant (adjusted hazard ratio 1.74, 95% confidence interval 1.00–3.00, *P* = .048), whereas broad FIGO stage II was not independently associated with mortality in this provisional reanalysis (Table 5).

**Table 5:** Univariate and multivariate Cox regression analysis for OS.

Variables	Univariate Analysis		Multivariate Analysis	
	HR (95% CI)	*P* Value	HR (95% CI)	*P* Value
Peritoneal washing cytology positive	2.27 (1.29–3.99),	.004	2.20 (1.25–3.87)	.007
FIGO stage (II vs I)	1.26 (0.73–2.17),	.408	1.30 (0.75–2.24)	.348
Lymph node metastasis	1.84 (1.07–3.16),	.029	1.74 (1.00–3.00)	.048
Deep myometrial invasion	0.97 (0.57–1.67)	.921	–	–
LVSI positive	0.77 (0.44–1.35)	.360	–	–
Tumor diameter ≥ 4 cm	0.79 (0.46–1.35)	.390	–	–
Preoperative SCC ≥ 1.5ng/mL	1.18 (0.62–2.25)	.614	–	–

### 3.6. Exploratory lymph-node metastasis model

The transparent two-biomarker logistic model was logit(P) = −5.3419 + 0.02510 × serum vascular endothelial growth factor + 0.02102 × tissue matrix metalloproteinase-9. Its area under the receiver operating characteristic curve was 0.774 (bootstrap 95% confidence interval 0.681–0.857). The numerical area under the curve exceeded that for either biomarker alone, but paired bootstrap comparisons did not establish statistical superiority (two-biomarker model vs vascular endothelial growth factor: difference approximately 0.049, 95% bootstrap confidence interval − 0.020 to 0.120, P ≈ 0.17; vs matrix metalloproteinase-9: difference approximately 0.061, 95% bootstrap confidence interval − 0.009 to 0.132, P ≈ 0.09).

Adding cytology status produced a coefficient of 0.1716 for cytology (*P* = .724) and essentially unchanged discrimination (area under the curve 0.774) (Table 6).

**Table 6:** ROC curve analysis of VEGF and MMP-9 for LNM diagnosis.

Biomarker	AUC (95% CI)	Optimal cutoff	Sensitivity (%)	Specificity (%)	Youden index
VEGF	0.725 (0.629–0.810)	89.40	66.7% (51.6–79.0)	74.4% (63.7–82.7)	0.410
Tissue MMP-9	0.713 (0.613–0.806)	109.80	73.8% (58.9–84.7)	60.3% (49.2–70.4)	0.341
Two-biomarker model	0.774 (0.681–0.857)	0.274	78.6% (64.1–88.3)	64.1% (53.0–73.9)	0.427
Two biomarkers + cytology sensitivity model	0.774 (0.679–0.857)	0.266	78.6% (64.1–88.3)	64.1% (53.0–73.9)	0.427

## 4. Discussion

This retrospective cohort study systematically investigated the association between peritoneal cytology status and survival outcomes in cervical cancer, alongside its potential diagnostic value for pelvic lymph node metastasis (LNM). Our data demonstrate that patients with positive peritoneal cytology (Cyt^+^) exhibit significantly reduced 5-year overall survival (OS) and progression-free survival (PFS), higher recurrence rates – particularly distant metastasis – and suggest an adverse prognostic association (adjusted HR = 2.15, *P* = .004) independent of FIGO stage and lymph node metastasis. Concomitant elevations in serum vascular endothelial growth factor (VEGF) and tissue matrix metalloproteinase-9 (MMP-9) levels among Cyt^+^ patients indicate a plausible biological link between intraperitoneal tumor cells and activation of metastatic pathways involving angiogenesis and extracellular matrix degradation. For LNM detection, the combination of VEGF and MMP-9 biomarkers showed improved diagnostic performance (AUC = 0.832, sensitivity 83.3%, specificity 80.8%) compared to individual markers. While promising, this approach requires external validation before clinical implementation.

### 4.1. Peritoneal cytology positivity as an independent prognostic factor

Kaplan–Meier analysis demonstrated poorer OS and PFS among patients with positive peritoneal washing cytology. However, because the median follow-up was shorter than 60 months and the number of patients remaining at risk at the 5-year time point was limited, the 5-year survival estimates should be interpreted cautiously. Longer follow-up in larger cohorts is required to obtain more precise estimates of long-term survival. This aligns with cross-cancer consensus: in ovarian cancer, positive peritoneal cytology defines FIGO IIIC stage and reduces 5-year survival by 28% ^[17]^; in endometrial cancer, it shortens recurrence-free survival by 14 months. ^[18]^ However, evidence in cervical cancer remains limited. A single-center study by Takigawa et al reported only a univariate association with recurrence-free survival (HR = 1.87, *P* = .043). ^[19]^ By rigorous baseline matching (balanced FIGO stage/tumor diameter, *P* > .05) and multivariate adjustment (7 covariates), our study establishes Cyt^+^ as an independent prognostic indicator, addressing biological gaps in FIGO staging. Mechanistically, free intraperitoneal malignant cells signify acquired peritoneal dissemination capacity. Elevated serum VEGF (98.25 vs72.83 ng/mL, *P* < .001) and tissue MMP-9 (142.60 vs 102.40 pg/mg, *P* < .001) in Cyt^+^ patients mirror the “MMP-9/VEGF pro-metastatic loop” in ovarian cancer.^[20]^ VEGF promotes intravasation via angiogenesis, while MMP-9 degrades basement membranes to enable invasion. Notably, distant recurrence predominated in Cyt^+^ patients (36.84% vs 10.71%, 3.4-fold increase) without pelvic recurrence differences, suggesting hematogenous spread via portal or thoracic ducts – consistent with molecular tracing studies where 76% of liver metastases shared TP53 mutations with primary tumors.^[21, 22]^

### 4.2. Molecular links between peritoneal cytology and invasive tumor microenvironment

Our findings are broadly consistent with previous cervical-cancer-specific studies showing associations of VEGF and MMP-9 with invasive clinicopathological characteristics and lymph-node metastasis. Increased VEGF may reflect enhanced tumor angiogenesis, whereas MMP-9 expression may reflect increased extracellular-matrix remodeling and invasive potential. Nevertheless, the present study demonstrates associations rather than mechanistic interactions. Although VEGF and MMP-9 may participate in interconnected biological processes during tumor progression, our data do not establish a direct VEGF–MMP-9 regulatory pathway or amplification loop. Further experimental studies are required to clarify the molecular interactions between these biomarkers in cervical cancer. ^[23]^ The two-biomarker model showed promising discrimination for lymph-node metastasis in the present dataset. However, because model development and performance assessment were conducted in the same relatively small cohort, the apparent AUC is likely subject to optimism. These findings should therefore be regarded as exploratory rather than as evidence for immediate clinical utility. Internal validation and, importantly, independent external validation are required before the model can be considered for clinical risk stratification.

Similarly, the observed associations between cytology status, serum VEGF, and tissue MMP-9 do not establish a directional or causal biological mechanism. Because the biomarkers were measured at a single time point in an observational study, the findings are best interpreted as associations consistent with – but not proof of – interactions between angiogenic and extracellular-matrix-remodeling processes.

### 4.3. Diagnostic integration of biomarkers with peritoneal cytology

The superior diagnostic performance of the combined VEGF/MMP-9 model (AUC = 0.832, sensitivity 83.3%, specificity 80.8%) for PLNM detection substantiates 2 critical pathophysiological concepts: Metastatic Cascade Synergy: The AUC enhancement from 0.762 (VEGF) and 0.718 (MMP-9) to 0.832 in the combined model (*P* < .05 vs individual markers) reflects the biological interdependence of angiogenesis and extracellular matrix remodeling in lymphatic dissemination. VEGF promotes endothelial permeability for tumor cell intravasation, while MMP-9 degrades basement membrane collagen IV, creating a coordinated “invasion- metastasis axis.”^[24]^ Peritoneal-Lymphatic Crosstalk: The significant VEGF/MMP-9 elevation in Cyt^+^ patients (median VEGF: 98.25 vs 72.83 ng/mL; MMP-9: 142.60 vs 102.40 pg/mg; *P* < .001) suggests peritoneal tumor cells actively prime metastatic niches. In cervical cancer, positive peritoneal washing cytology may represent the presence of exfoliated malignant cells within the peritoneal cavity and has been associated with adverse clinicopathological characteristics in previous studies. However, the biological route by which cervical tumor cells reach the peritoneal cavity remains incompletely understood.^[25,26]^ In the multivariable analysis, dual VEGF/MMP-9 positivity was associated with an increased likelihood of pelvic lymph-node metastasis (OR, 3.76; 95% CI, 1.72–8.23; *P* = .001). This association should be interpreted as exploratory because the biomarker model was developed in a single cohort and has not yet undergone external validation.

## 5. Controversies and limitations

The present findings showed that higher VEGF levels and MMP-9 expression were associated with positive peritoneal washing cytology and more aggressive clinicopathological characteristics. These findings may reflect the coexistence of enhanced angiogenesis, extracellular-matrix remodeling, and tumor invasiveness. Previous experimental studies have suggested potential biological interactions between VEGF-related angiogenic signaling and MMP-9-mediated matrix degradation. However, the present study was observational and did not include mechanistic experiments, pathway inhibition, functional validation, or longitudinal biomarker measurements. Therefore, our findings cannot establish a directional or causal “MMP-9/VEGF loop,” nor can they determine whether VEGF directly regulates MMP-9 or vice versa. The observed association should instead be interpreted as hypothesis-generating evidence that VEGF and MMP-9 may jointly reflect an aggressive tumor phenotype. Further experimental studies are warranted to clarify the molecular relationship between these biomarkers and their potential roles in tumor-cell dissemination and lymphatic metastasis.

This study has several important limitations. First, the retrospective observational design is inherently susceptible to information bias, selection bias, and residual confounding. In particular, patient inclusion depended on the availability of complete clinicopathological, biomarker, cytological, and follow-up data, which may have resulted in a selected study population and limited the representativeness of the cohort. Second, although multivariable analyses were performed to account for clinically relevant prognostic factors, unmeasured or incompletely recorded variables may still have influenced the observed associations. Third, differences in surgical procedures and postoperative adjuvant treatments were not fully controlled, and such treatment heterogeneity may have affected recurrence and survival outcomes; therefore, residual treatment-related confounding cannot be excluded. Fourth, this was a single-center study with a relatively small sample size and a limited number of outcome events, which may reduce the precision and stability of multivariable estimates and increase the risk of model overfitting. Fifth, VEGF and MMP-9 were assessed at a single time point, precluding evaluation of temporal changes and causal relationships. Finally, the study population consisted predominantly of surgically treated patients with early-stage cervical cancer, which may limit the generalizability of the findings to patients with advanced-stage disease or those receiving nonsurgical treatment. An additional limitation is that the study population was restricted to surgically treated patients with FIGO stage I–II cervical cancer, and no patients with stage III–IV disease were included. Therefore, the present findings may not be generalizable to patients with locally advanced or metastatic cervical cancer, who typically have different tumor biology, treatment strategies, and prognostic profiles. In particular, the prognostic value of peritoneal washing cytology and the associations of VEGF and MMP-9 observed in this cohort should not be extrapolated to advanced-stage disease without further validation. Future studies should include broader FIGO stage distributions and prospectively evaluate these biomarkers in patients receiving definitive chemoradiotherapy or systemic treatment. The multivariable model included 7 candidate variables with 53 outcome events, yielding an events-per-variable ratio of approximately 7.6. This modest EPV may increase the risk of overfitting and coefficient instability. Therefore, the adjusted hazard ratios should be interpreted as exploratory estimates. The consistency of the findings in bootstrap and parsimonious sensitivity analyses provides some reassurance, but external validation in larger cohorts is required. These limitations should be considered when interpreting the present results. Larger prospective multicenter studies with standardized treatment strategies, longitudinal biomarker assessment, and more comprehensive control of potential confounding factors are required to validate these findings.

In this retrospective cohort of surgically treated cervical cancer patients, positive peritoneal washing cytology was associated with poorer survival and a higher frequency of distant recurrence. Cytology-positive patients also had higher serum VEGF and tissue MMP-9 levels, although these cross-sectional associations do not establish a causal biological mechanism. The combined VEGF/MMP-9 model demonstrated exploratory discrimination for lymph-node metastasis but was developed in the same single-center cohort and has not been externally validated. Larger multicenter studies with standardized biomarker assessment, detailed FIGO staging, and independent model validation are required to confirm these findings and determine their potential clinical relevance.

## Author contributions

**Conceptualization:** Qianwen Zhang, Tao Wang.

**Data curation:** Qianwen Zhang, Xiaoqin Li, Qi Wang, Zhiqian Lin.

**Formal analysis:** Qianwen Zhang, Xiaoqin Li, Zhiqian Lin.

**Funding acquisition:** Tao Wang, Xiaoqin Li.

**Investigation:** Qianwen Zhang, Xiaoqin Li, Qi Wang.

**Supervision:** Tao Wang.

**Writing – original draft:** Qianwen Zhang.

**Writing – review & editing:** Qianwen Zhang, Tao Wang.
